# Canine Distemper Virus (CDV) Transit Through the Americas: Need to Assess the Impact of CDV Infection on Species Conservation

**DOI:** 10.3389/fmicb.2020.00810

**Published:** 2020-05-21

**Authors:** Santiago Rendon-Marin, Marlen Martinez-Gutierrez, José Antonio Suarez, Julian Ruiz-Saenz

**Affiliations:** ^1^Grupo de Investigación en Ciencias Animales—GRICA, Universidad Cooperativa de Colombia, Bucaramanga, Colombia; ^2^Infettare, Facultad de Medicina, Universidad Cooperativa de Colombia, Medellín, Colombia; ^3^Investigador SNI Senacyt Panamá, Clinical Research Deparment, Instituto Conmemorativo Gorgas de Estudios de la Salud, Panama City, Panama; ^4^Asociación Colombiana de Virología, Bogotá, Colombia

**Keywords:** canine distemper, conservation, interspecies transmission, morbillivirus, wildlife corridors

*Canine Morbillivirus* (also known as Canine Distemper Virus—CDV) is the causative agent of one of the most important diseases in domestic dogs and wild fauna. It belongs to the *Paramyxoviridae* family, order *Mononegavirales*, which has a non-segmented, single-stranded linear negative polarity RNA genome (Lamb and Parks, 2013). The clinical disease is characterized by moderate to severe respiratory signs, gastrointestinal issues, immune suppression, and/or neurological disease (De Vries et al., 2015; Pfeffermann et al., 2018).

Beyond domestic dogs (*Canis lupus familiaris*), CDV infects multiple species within the Order Carnivora, including several wild species as *Canidae, Felidae, Procyonidae, Mustelidae, Hyaenidae, Ursidae*, and *Viverridae* families. CDV infection has also been reported in other Orders such as Rodentia (rodents), Primates, Artiodactyla, and Proboscidea (Martinez-Gutierrez and Ruiz-Saenz, 2016). More recently, CDV has been confirmed as the etiological agent of clinical illness in two different families of the Order Pilosa in South America and in one in North America. The first case was reported on a captive southern tamandua (*Tamandua tetradactyla*) with neurological signs (Lunardi et al., 2018). The other case was described in a young giant anteater, showing prostration, nasal, and eye discharge (*Myrmecophaga tridactyla*). Both of these belong to the *Myrmecophagidae* Family (Debesa Belizario Granjeiro et al., 2020). Also, in the USA, a Linnaeus's 2-toed sloth (*Choloepus didactylus*), family *Choloepodidae*, order Pilosa, was reported with clinical signs that included hyporexia, lethargy, mucopurulent nasal discharge, and oral and facial ulcers confirmed as CDV South America/North America-4 lineage (Watson et al., 2020). However, the complete distribution of different CDV strains and Lineages among wildlife species throughout the Americas have not been thoroughly investigated.

Phylogenetic analysis has demonstrated the circulation of a new CDV lineage in the Americas. Classified as “South America/North America-4” due to its intercontinental distribution and the monophyletic grouping, strains have been isolated in dogs from Ecuador and Colombia (South America) and wild and domestic dogs in the United States (North America) (Duque-Valencia et al., 2019a). As plylogeography confirmed, the “South America/North America-4” lineage circulated first in Colombia and Ecuador, then in the United States, and again in Colombia [see Figures 3, 4 in Duque-Valencia et al. (2019a)]. It has been stated that the uncontrolled commercialization of puppies from South America to the United States could be the route of transmission of the “South America/North America-4” lineage among these two continent regions; the role of wildlife in virus dissemination throughout the entire continent can also not be ruled out.

In this context, it is important to understand the impact of the intercontinental transmission of CDV, since the implementation of control policies should cross borders and generate unified international policies that prevent the presentation of cases in domestic and wild fauna. This type of regional disease control program has proven to face multiple challenges in political interaction between countries and requires the leadership of multilateral entities to help unify criteria and policies (Hukic et al., 2010; Sleeman et al., 2017).

On the other hand, wildlife corridors are connections across the landscape that link up areas of habitats, which supports natural processes, including the movement of species to find resources such as food and water (Chetkiewicz et al., 2006). Corridors are species and process specific (e.g., migration and dispersal). They do not necessarily consist of breeding habitats, but rather are intended to provide connectivity between habitat patches (Beier et al., 2008). Different corridors have been designed to protect wild fauna among the Americas. The Mesoamerican Biological Corridor (Holland, 2012) or the Jaguar Corridor Initiative (Zeller et al., 2013) are a wide range of initiatives to protect species and habitats through maintaining or enhancing connectivity between populations, which contributes to the survival of the species by allowing the dispersion of individuals from their native ranges to new territories, enabling the exchange of genetic material among different isolated populations (Zeller et al., 2013).

However, it has been postulated that wildlife corridors may be at risk of contracting diseases through interaction with domestic and livestock fauna (Grootenhuis, 2000). Moreover, it is well known that viral diseases could have a negative role in animal conservation. For example, highly contagious diseases with high mortality rates, such as CDV and rinderpest, have decimated wildlife populations in the past (Loots et al., 2017). It is possible that diverse pathogens may have negative effects on population health due to reconnecting fragmented habitats, which could cause pathogen invasions and would result in a negative effect on species conservation (Hess, 1994).

Previously, the main role of Panamá for the movement and gene flow of numerous neotropical forest species has been described (Leigh et al., 2014), mainly due to the geographical location in the Mesoamerican Biological Corridor. Moreover, recent assessments have reported that the Atlantic side of the isthmus is critical for the occupancy and connectivity of important mammal species. These include ungulates such as the Baird's tapir (*Tapirus bairdii*), white-lipped peccary (*Tayassu pecari*), collared peccary (*Pecari tajacu*), white-tailed deer (*Odocoileus virginianus*) and the Central American red brocket deer (*Mazama temama*), carnivores such as the jaguar (*Panthera onca*), puma (*Puma concolor*), and ocelot (*Leopardus pardalis*), and insectivores like the giant anteater (*Myrmecophaga tridactyla*); all of them are mostly forest specialists (Meyer et al., 2020a,b).

The mentioned list includes a vast array of large and medium-sized mammals (both carnivorous and non-carnivorous) ranging from ungulates to carnivores and even insectivorous species, and CDV has been reported to infect most of those species (Martinez-Gutierrez and Ruiz-Saenz, 2016). In fact, the reported case of a giant anteater showing clinical illness confirmed to be canine distemper (Debesa Belizario Granjeiro et al., 2020) could just represent the tip of the iceberg, relative to the total number of infections in the wild. When we analyse the connectivity of the Mesoamerican Biological Corridor or the Jaguar Corridor in the Americas, we could observe the successful bridge between central and South America (Figure 1). However, this same bridge could possibly be used as a transmission bridge for “Multi-Host viruses” such as the CDV (Duque-Valencia et al., 2019b). A similar phenomena has been described for other important viruses such as the West Nile Virus and the Avian Influenza Virus that efficiently use migratory routes, leading to dissemination and intercontinental transmission of viruses in animal populations (Lee et al., 2015; Afanador-Villamizar et al., 2017; Kramer et al., 2019).

**Figure 1:**
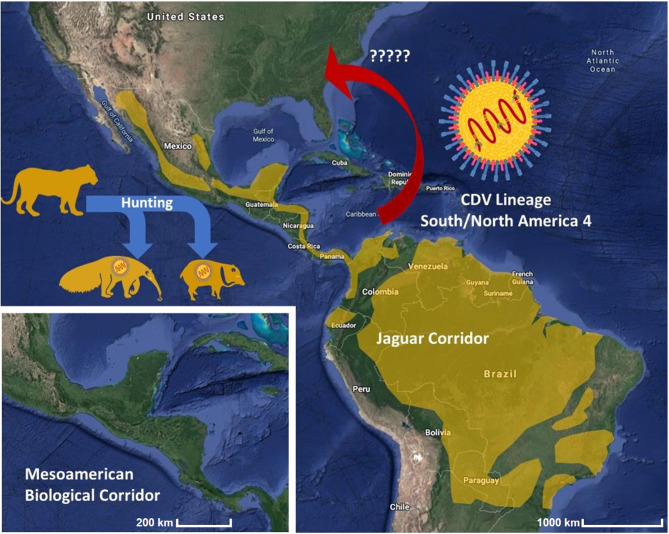
Schematic representation of the possible transmission route of CDV trough the biological corridors. Box below highlights the Mesoamerican Biological Corridor. Yellow shadow highlights the Jaguar Corridor. See text for references.

The continuous reports of CDV infection in wild felids and endangered species such as the Anteater from central and South America has raised a concern over the critical role of CDV in big cat conservation in the Americas. The same occurred with wild Siberian tigers (*Panthera tigris altaica*) (Gilbert et al., 2014, 2015; Zhang et al., 2017) and Giant pandas (*Ailuropoda melanoleuca*) (Feng et al., 2016; Jin et al., 2017; Zhao et al., 2017) in which the risk of extinction of the species associated with CDV infection has been characterized.

In the Mesoamerican and South American areas, CDV has been described as infecting the cougar (*Puma concolor*), margay (*Leopardus wiedii*), jaguarundi (*Herpailurus yagouaroundi*), Jaguars (*Panther onca*), ocelots (*Leopardus pardalis*), jaguarundis (*Puma yaguaroundi*), pampas cat (*Leopardus colocolo*), and other wild canids such as the maned wolf (*Chrysocyon brachyurus*), crab-eating fox (*Cerdocyon thous*), hoary fox (*Pseudalopex vetulus*), striped hog-nosed skunk (*Conepatus semistriatus*), and coati (*Nasua nasua*), among others (Avendano et al., 2016; Furtado et al., 2016; Viana et al., 2020). The animals mentioned above such as cougars, jaguars, and other medium and big carnivorous often prey on livestock and native prey including mesocarnivorous and medium-sized mammals such as anteaters and tamanduas (Cavalcanti and Gese, 2010) which could be shown to be a new reservoir for CDV. In fact, it has been described that Giant anteaters (*Myrmecophaga tridactyla*) contribute more than 75 % of biomass to the observed diet of the jaguar (*Panthera onca*) in the Cerrado, central Brazil (Sollmann et al., 2013). Also, it has been shown that 21% of the jaguar diet includes peccaries (mostly *Tayassu pecari*), another species that has been commonly been reported as being at risk of infection with CDV (Noon et al., 2003), suggesting the imminent risk in those species to get infected with a multi-host viral pathogen such as CDV when they hunt their prey.

## Concluding Remarks

Our understanding of the circulation of CDV in the Americas allows us to speculate on the number of critical factors. These factors include the presence of specific viral lineages in large geographical areas, and the susceptibility of circulating species in interconnected regions which favors viral exchange across the continent and represents a risk for wild endangered populations. For this reason, it is imperative to establish not only the dynamics but also diverse aspects of the circulation of CDV among wildlife in the Americas, such as prevalent linages and their associations that would enable us to elucidate the main circulating stains.

There is a great possibility that there is an underreporting of CDV infection and that the published cases only represent the tip of the iceberg in the epidemiology of CDV in wild populations in the Americas.

We recommend enhancing the monitoring of CDV among wildlife corridors and to evaluate the CDV dynamics among different target populations regardless of whether clinical signs are observed. Moreover, we encourage the implementation of an interdisciplinary approach that would enable us to understand the critical role of high impact/mortality diseases such as CDV on the wildlife conservation. Also we encourage the assessment of the impact of CDV circulation and vaccine coverage of domestic dogs on wildlife epidemics, as this topic has not been evaluated in the America corridor areas.

## Author Contributions

JR-S conceived the study. SR-M, MM-G, JS, and JR-S were involved in all other aspects of the study, including data collection, data analysis, drafting and editing the paper. All authors read and approved the final manuscript.

## Conflict of Interest

The authors declare that the research was conducted in the absence of any commercial or financial relationships that could be construed as a potential conflict of interest.
